# Structural insights into 1,4-bis­(neopent­yloxy)pillar[5]arene and the pyridine host–guest system

**DOI:** 10.1107/S2414314624010733

**Published:** 2024-11-08

**Authors:** Mickey Vinodh, Fatemeh H. Alipour, Talal F. Al-Azemi

**Affiliations:** a Department of Chemistry, Kuwait University, PO Box 5969, Safat 13060, Kuwait; Goethe-Universität Frankfurt, Germany

**Keywords:** crystal structure, host–guest system, pentyl­oxypillar[5]arene, pyridine guest

## Abstract

The crystal structure of 1,4-bis­(neopent­yloxy)pillar[5]arene, C_95_H_140_N_2_O_10_ (**TbuP)**, featuring two encapsulated pyridine mol­ecules, reveals significant host–guest inter­actions. Inter­estingly, the pyridine guests are positioned near the neopent­yloxy substituents instead of the electron-rich aromatic core of the pillar[5]arene. This spatial arrangement suggests a preference for the pyridine mol­ecules to engage with the aliphatic regions of the host. Detailed analysis of the structural characteristics of this host–guest system (**TbuP·2Py)**, as well as its packing pattern within the crystal network, is presented and discussed.

## Abstract 2

The crystal structure of a neo­pentyl­oxypillar[5]arene with two pyridine mol­ecules encapsulated in the macrocyclic cavity is reported.

## Graphical abstract

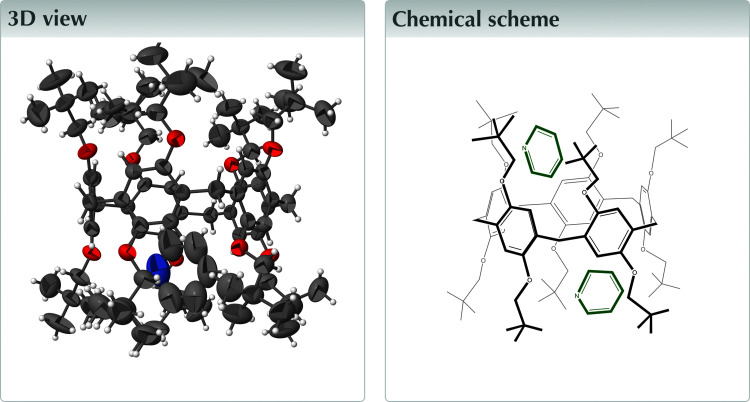

## Structure description

The intrinsic cavity of pillar[*n*]arenes exhibits fascinating guest-encapsulation behavior, significantly influenced by their peripheral substitutions (Ogoshi *et al.*, 2016 ▸; Li *et al.*, 2020 ▸). The electron-rich cavity of pillar[5]arene tends to favor the inclusion of long aliphatic chains over aromatic guests. This limitation in encapsulating aromatic guests restricts the applications of pillararenes as functional materials. Optimizing the peripheral substituents on pillar[5]arene is therefore crucial to enhance its encapsulation performance for aromatic mol­ecules. By carefully selecting these substituents, the inter­actions and selectivity for specific aromatic guests can be fine-tuned, making pillar[5]arenes valuable for various applications, including separation technologies and sensor design (Jie *et al.*, 2018 ▸; Wu *et al.*, 2020 ▸; Wu, Wu, Li *et al.*, 2023 ▸; Wu, Tang *et al.*, 2023 ▸; Zhao *et al.*, 2024 ▸).

**Figure 1:**
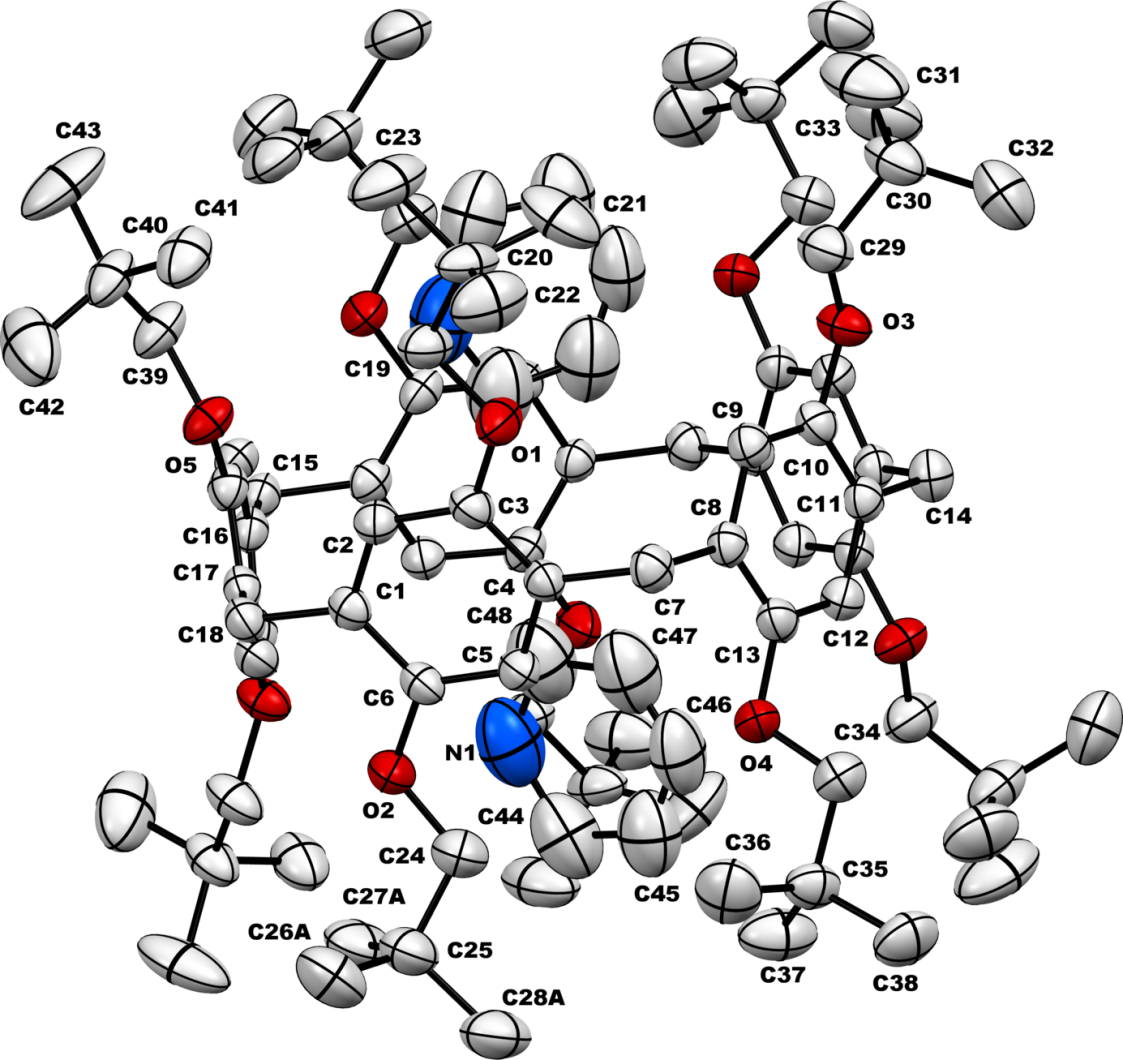
Symmetry-expanded crystal structure of **TBuP·2Py** with displacement ellipsoids (30% probability; only the symmetry independent atoms are labeled). Only the major components of the disordered moieties are shown. Hydrogen atoms are omitted for clarity.

**Figure 2:**
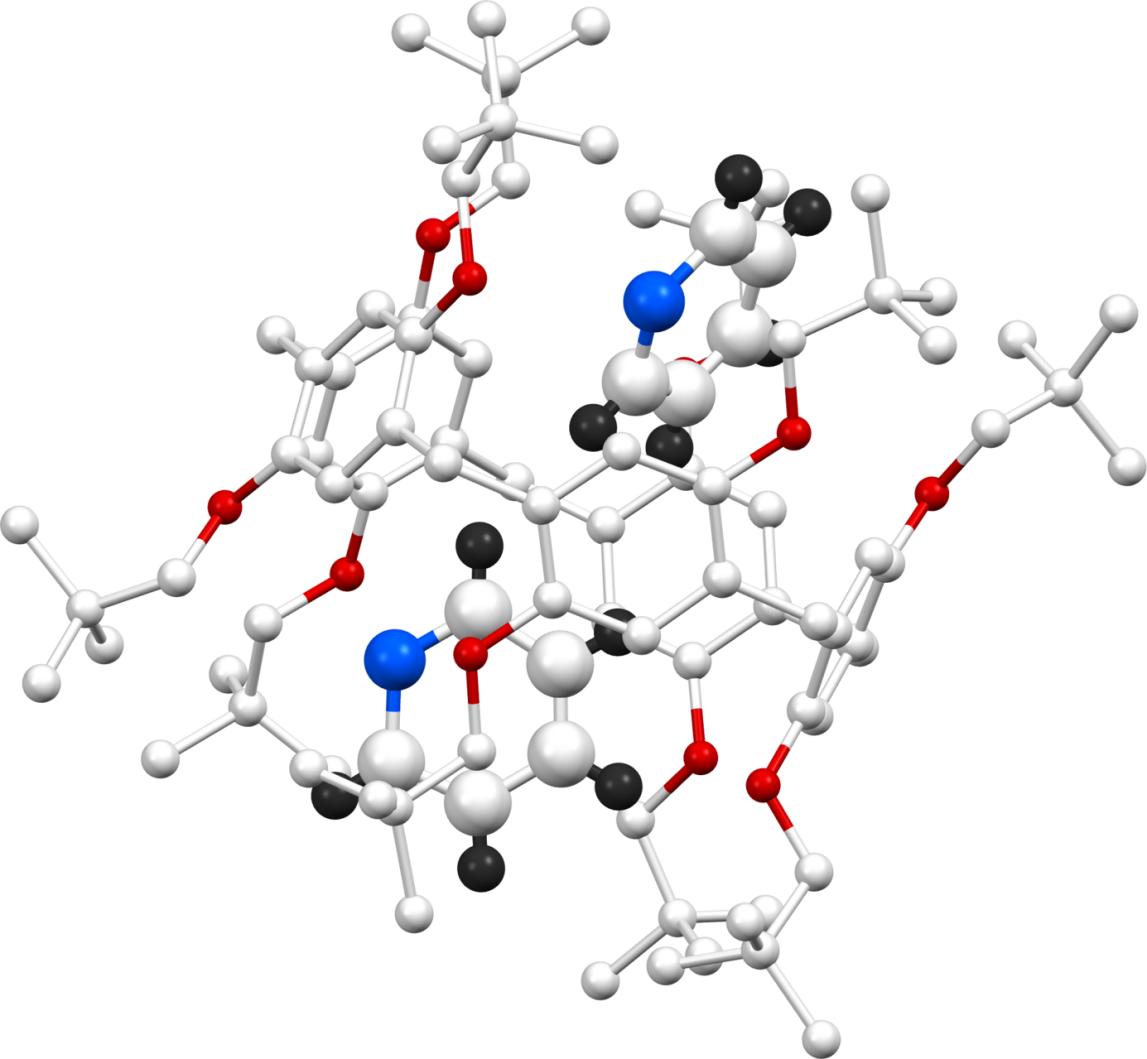
Crystal structure (symmetry expanded) of **TBuP·2Py**. Only the major components of the disordered moieties are shown. Hydrogen atoms belonging to the pillar[5]arene are omitted for clarity.

**Figure 3:**
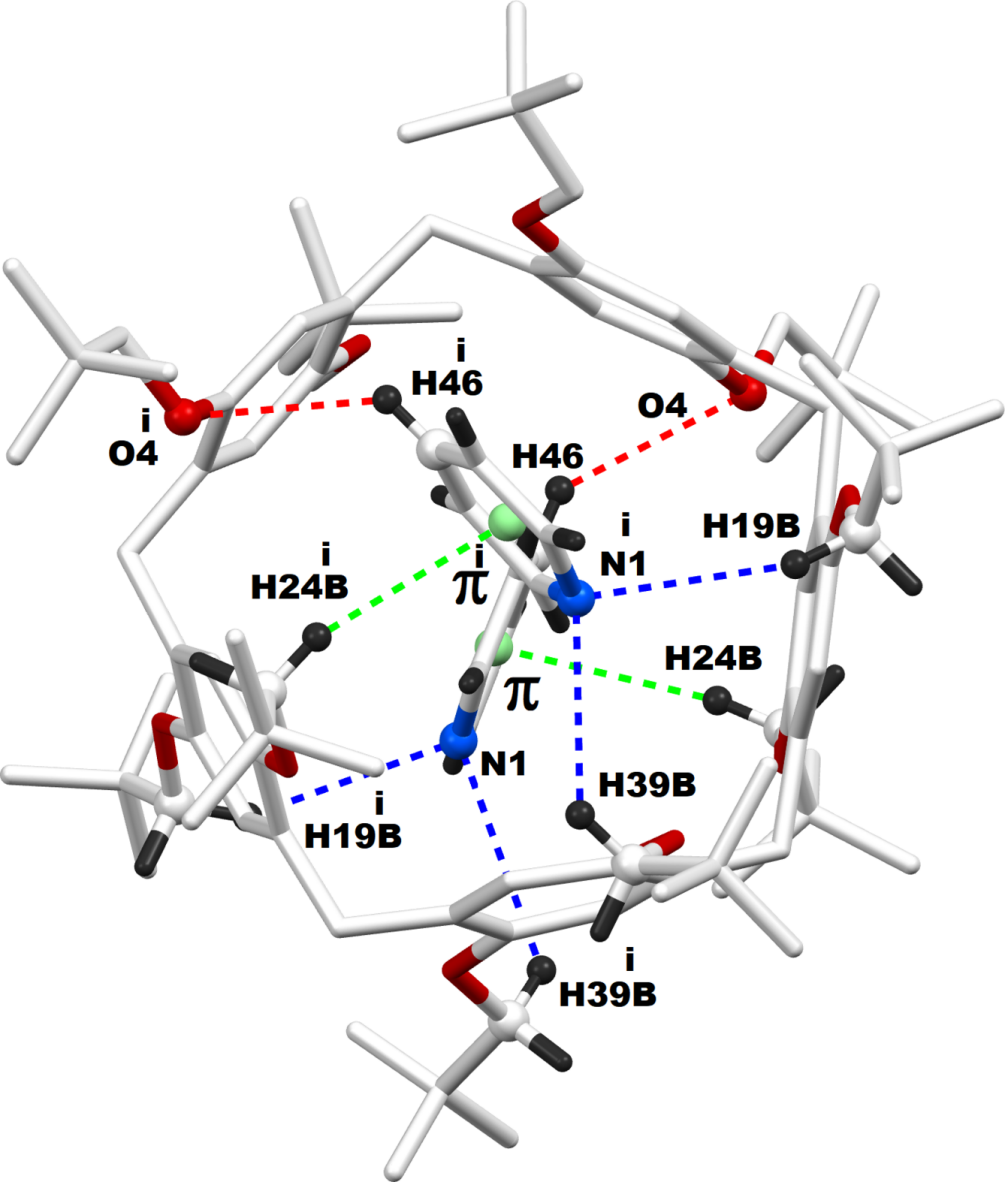
Supra­molecular inter­actions between the pillar[5]arene host and pyridine guests. π denotes the centroid of the pyridine ring. Symmetry code: (i) 1 − *x*, −*y*, *z.*

The title inclusion complex (**TbuP·2Py)** crystallizes in the ortho­rhom­bic crystal system, space group *Fdd*2. The asymmetric unit contains half of the pillar[5]arene mol­ecule (Fig. 1 ▸), with the complete structure revealed through symmetry expansion (Fig. 2 ▸). This pillar[5]arene exhibits a penta­gonal macrocyclic shape with neopent­yloxy substitutions at both the top and bottom rims. Notably, one of the neopent­yloxy substituents shows positional disorder within the crystal, with only the higher occupancy orientation shown. In the crystal structure of the **TbuP·2Py** system, two pyridine mol­ecules are encapsulated within the cavity of the pillar[5]arene. These pyridine guests are strategically positioned adjacent to the neopent­yloxy regions rather than the electron-rich core, enhancing stability through non-bonding inter­actions. This is illustrated in the Fig. 3 ▸, with qu­anti­tative details provided in the accompanying table (Table 1 ▸).

**Figure 4:**
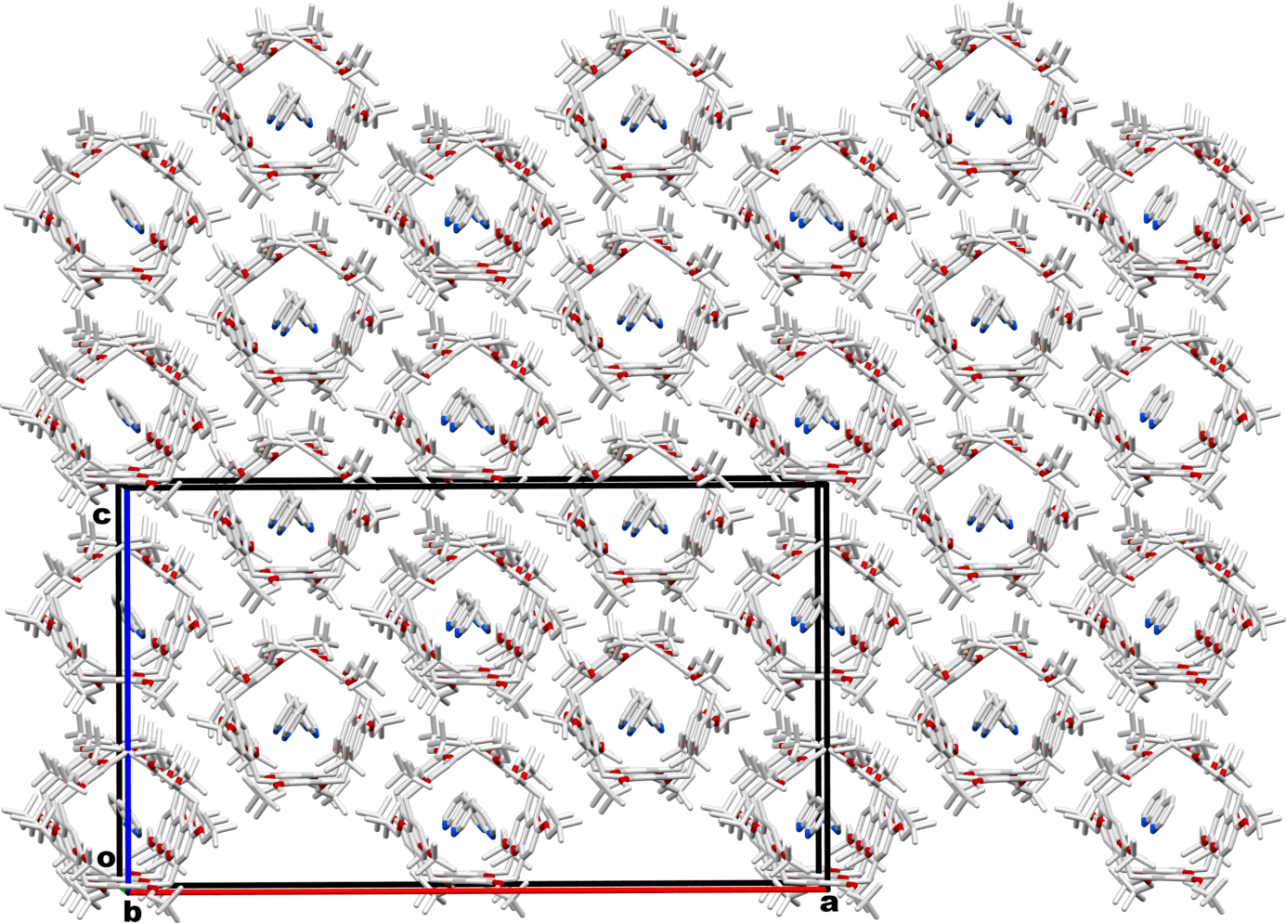
Packing pattern of **TBuP·2Py** system in the crystal network showing one-dimensional channels along the *b*-axis direction.

The neopent­yloxy substitutions on the rims of the pillar[5]arene play a crucial role in accommodating aromatic guests within the intrinsic cavity, which typically favors non-aromatic linear mol­ecules. The **TbuP·2Py** system exhibits a unique packing arrangement, forming one-dimensional channels along the *b*-axis direction in the crystal network (Fig. 4 ▸). This specific organization is crucial for facilitating guest encapsulation and removal. Such arrangements enable the use of pillar[5]arene-based nonporous adaptive crystals (NACs) in adsorption and separation processes, highlighting the potential for tuning material properties based on guest inter­actions and opening avenues for further research and practical applications.

## Synthesis and crystallization

The synthesis and characterization of 1,2,3,4,5-(1,4-neopent­yloxy)pillar[5]arene (**TBuP**) have been previously described (Al-Azemi *et al.*, 2019 ▸). Colorless blocks of **TBuP·2Py** crystals, suitable for single-crystal analysis, were obtained by dissolving 25 mg of **TBuP** in a chloro­form: pyridine solvent mixture (90:10 *v*/*v*, 1 ml) and allowing for slow solvent evaporation.

## Refinement

Crystal data, data collection and structure refinement details are summarized in Table 2 ▸. One of the neopentyl spacers of the pillar[5]arene in the crystal was found to be disordered. The most satisfactory occupancies for these disordered neopent­yloxy fractions were 0.607 (17):0.393 (17) for the major and minor components, respectively. The DELU and SIMU commands were used in the refinement to restrain the displacement factors of these disordered components. Additionally, the DELU and SIMU commands were used to restrain the displacement factors of the atoms belonging to the guest pyridine and another neopentyl moiety of the pillar[5]arene.

## Supplementary Material

Crystal structure: contains datablock(s) I. DOI: 10.1107/S2414314624010733/bt4159sup1.cif

Structure factors: contains datablock(s) I. DOI: 10.1107/S2414314624010733/bt4159Isup2.hkl

Supporting information file. DOI: 10.1107/S2414314624010733/bt4159Isup3.mol

CCDC reference: 2393384

Additional supporting information:  crystallographic information; 3D view; checkCIF report

## Figures and Tables

**Table 1:** Hydrogen-bond geometry (Å, °) π denotes the centroid of the pyridine ring.

*D*—H⋯*A*	*D*—H	H⋯*A*	*D*⋯*A*	*D*—H⋯*A*
C46—H46⋯O4	0.93	2.88	3.56 (1)	131
C24—H24*B*⋯π	0.97	2.99	3.921 (11)	161
C19—H19*B*⋯N1^i^	0.97	2.97	3.81 (1)	145
C39—H39*B*⋯N1^i^	0.97	3.17	3.74 (1)	120

Symmetry code: (i) 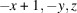.

**Table 2:** Experimental details

Crystal data	
Chemical formula	C_95_H_140_N_2_O_10_
*M*_r_	1470.08
Crystal system, space group	Orthorhombic, *Fdd*2
Temperature (K)	293
*a*, *b*, *c* (Å)	47.848 (5), 14.2452 (14), 27.679 (2)
*V* (Å^3^)	18866 (3)
*Z*	8
Radiation type	Mo *K*α
μ (mm^−1^)	0.07
Crystal size (mm)	0.17 × 0.14 × 0.11

Data collection	
Diffractometer	Rigaku R-AXIS RAPID
Absorption correction	Multi-scan (*ABSCOR*; Higashi, 1995 ▸)
*T*_min_, *T*_max_	0.156, 0.991
No. of measured, independent and observed [*I* > 2σ(*I*)] reflections	23011, 7942, 3988
*R*_int_	0.087
(sin θ/λ)_max_ (Å^−1^)	0.595

Refinement	
*R*[*F*^2^ > 2σ(*F*^2^)], *wR*(*F*^2^), *S*	0.068, 0.192, 0.99
No. of reflections	7942
No. of parameters	526
No. of restraints	267
H-atom treatment	H-atom parameters constrained
Δρ_max_, Δρ_min_ (e Å^−3^)	0.25, −0.16

Computer programs: *CrystalClear* (Rigaku, 2016 ▸), *CrystalStructure* (Rigaku, 2017 ▸), *SHELXL2019/3* (Sheldrick, 2015 ▸) and *Mercury* (Macrae *et al.*, 2020 ▸).

## full crystallographic data

### 1,4-Bis(neopentyloxy)pillar[5]arene Crystal data

**Table:**

C_95_H_140_N_2_O_10_	*D*_x_ = 1.035 Mg m^−^^3^
*M**_r_* = 1470.08	Mo *K*α radiation, λ = 0.71075 Å
Orthorhombic, *F**d**d*2	Cell parameters from 11195 reflections
*a* = 47.848 (5) Å	θ = 3.1–25.0°
*b* = 14.2452 (14) Å	µ = 0.07 mm^−^^1^
*c* = 27.679 (2) Å	*T* = 293 K
*V* = 18866 (3) Å^3^	Block, colorless
*Z* = 8	0.17 × 0.14 × 0.11 mm
*F*(000) = 6432	

### 1,4-Bis(neopentyloxy)pillar[5]arene Data collection

**Table:**

Rigaku R-AXIS RAPID diffractometer	3988 reflections with *I* > 2σ(*I*)
Detector resolution: 10.000 pixels mm^-1^	*R*_int_ = 0.087
ω scans	θ_max_ = 25.0°, θ_min_ = 3.2°
Absorption correction: multi-scan (ABSCOR; Higashi, 1995)	*h* = −56→54
*T*_min_ = 0.156, *T*_max_ = 0.991	*k* = −16→16
23011 measured reflections	*l* = −31→29
7942 independent reflections	

### 1,4-Bis(neopentyloxy)pillar[5]arene Refinement

**Table:**

Refinement on *F*^2^	267 restraints
Least-squares matrix: full	Hydrogen site location: inferred from neighbouring sites
*R*[*F*^2^ > 2σ(*F*^2^)] = 0.068	H-atom parameters constrained
*wR*(*F*^2^) = 0.192	*w* = 1/[σ^2^(*F*_o_^2^) + (0.0931*P*)^2^] where *P* = (*F*_o_^2^ + 2*F*_c_^2^)/3
*S* = 0.99	(Δ/σ)_max_ < 0.001
7942 reflections	Δρ_max_ = 0.25 e Å^−^^3^
526 parameters	Δρ_min_ = −0.16 e Å^−^^3^

### 1,4-Bis(neopentyloxy)pillar[5]arene Special details

**Table:**

Geometry. All esds (except the esd in the dihedral angle between two l.s. planes) are estimated using the full covariance matrix. The cell esds are taken into account individually in the estimation of esds in distances, angles and torsion angles; correlations between esds in cell parameters are only used when they are defined by crystal symmetry. An approximate (isotropic) treatment of cell esds is used for estimating esds involving l.s. planes.
Refinement. The single-crystal data collection was performed using a Rigaku Rapid II diffractometer with Mo*-Kα* radiation at 293 K. The data was processed using the '*CrystalClear*' software package. The structure was solved by direct methods using the '*CrystalStructure*' crystallographic software package, and refinement was carried out with *SHELXL2019*/3. All the hydrogen atoms were positioned geometrically, with C—H distances of methyl, methylene, and aromatic groups being 0.96, 0.97, and 0.93 Å, respectively, and the displacement factors of hydrogen atoms were set to *U_iso_*(H) = 1.2*U_eq_*(C), for hydrogen atoms of the methyl groups the displacement parameters were set to *U_iso_*(H) = 1.5*U_eq_*(C).

### 1,4-Bis(neopentyloxy)pillar[5]arene Fractional atomic coordinates and isotropic or equivalent isotropic displacement parameters (Å^2^)

**Table:**

	*x*	*y*	*z*	*U*_iso_*/*U*_eq_	Occ. (<1)
O1	0.60191 (11)	−0.1383 (3)	0.65859 (15)	0.0888 (15)	
N1	0.4746 (2)	0.2097 (11)	0.6285 (4)	0.180 (4)	
O2	0.56089 (11)	0.1905 (3)	0.58101 (15)	0.0846 (14)	
O3	0.52946 (10)	−0.1612 (3)	0.81964 (17)	0.0763 (12)	
O4	0.57102 (9)	0.1804 (3)	0.75905 (14)	0.0715 (12)	
O5	0.53741 (8)	−0.1469 (3)	0.51250 (16)	0.0715 (12)	
C1	0.57093 (11)	0.0302 (4)	0.5724 (2)	0.0562 (15)	
C2	0.58163 (13)	−0.0509 (4)	0.5915 (2)	0.0624 (16)	
H2	0.582871	−0.103606	0.571735	0.075*	
C3	0.59073 (13)	−0.0579 (5)	0.6391 (2)	0.0660 (17)	
C4	0.58896 (12)	0.0193 (4)	0.6688 (2)	0.0553 (15)	
C5	0.57858 (13)	0.1030 (4)	0.6500 (2)	0.0629 (17)	
H5	0.577422	0.155837	0.669658	0.076*	
C6	0.56995 (13)	0.1082 (4)	0.6022 (2)	0.0599 (16)	
C7	0.59780 (13)	0.0167 (5)	0.7218 (2)	0.0659 (18)	
H7A	0.609585	−0.037734	0.727080	0.079*	
H7B	0.608792	0.072201	0.728921	0.079*	
C8	0.57318 (12)	0.0125 (4)	0.75623 (19)	0.0578 (15)	
C9	0.56235 (13)	−0.0727 (4)	0.7712 (2)	0.0627 (16)	
H9	0.570529	−0.128118	0.760413	0.075*	
C10	0.53970 (14)	−0.0770 (4)	0.8017 (2)	0.0612 (16)	
C11	0.52636 (12)	0.0041 (5)	0.8179 (2)	0.0581 (15)	
C12	0.53756 (14)	0.0891 (5)	0.8030 (2)	0.0653 (17)	
H12	0.529136	0.144713	0.812916	0.078*	
C13	0.56101 (14)	0.0931 (5)	0.7737 (2)	0.0656 (16)	
C14	0.500000	0.000000	0.8479 (4)	0.072 (3)	
H144	0.4987 (16)	0.052 (5)	0.865 (3)	0.107*	
C15	0.48974 (14)	−0.0886 (4)	0.5176 (2)	0.0650 (16)	
H15	0.482886	−0.149562	0.519299	0.078*	
C16	0.51837 (14)	−0.0754 (5)	0.5155 (2)	0.0600 (15)	
C17	0.52911 (13)	0.0152 (4)	0.51732 (19)	0.0547 (15)	
C18	0.56011 (13)	0.0331 (4)	0.5206 (2)	0.0622 (16)	
H18A	0.564203	0.094187	0.506801	0.075*	
H18B	0.569905	−0.013711	0.501611	0.075*	
C19	0.60495 (17)	−0.2178 (5)	0.6288 (3)	0.085 (2)	
H19A	0.615767	−0.201267	0.600435	0.101*	
H19B	0.586672	−0.238811	0.618149	0.101*	
C20	0.61933 (17)	−0.2962 (5)	0.6554 (3)	0.090 (2)	
C21	0.6033 (3)	−0.3219 (8)	0.7008 (4)	0.166 (5)	
H21A	0.583828	−0.328691	0.693265	0.199*	
H21B	0.605613	−0.273314	0.724529	0.199*	
H21C	0.610332	−0.380082	0.713490	0.199*	
C22	0.64888 (19)	−0.2686 (6)	0.6669 (4)	0.137 (4)	
H22A	0.658865	−0.322241	0.678845	0.164*	
H22B	0.648845	−0.220143	0.690960	0.164*	
H22C	0.657826	−0.245838	0.638122	0.164*	
C23	0.6205 (3)	−0.3799 (6)	0.6225 (4)	0.150 (4)	
H23A	0.601810	−0.399755	0.614853	0.179*	
H23B	0.630241	−0.430146	0.638258	0.179*	
H23C	0.630090	−0.363319	0.593259	0.179*	
C24	0.5605 (2)	0.2736 (5)	0.6084 (3)	0.104 (2)	
H24A	0.576816	0.275167	0.629095	0.125*	
H24B	0.544059	0.273307	0.629037	0.125*	
C25	0.5600 (2)	0.3590 (6)	0.5781 (3)	0.104 (2)	
C26A	0.5842 (5)	0.3647 (14)	0.5425 (8)	0.140 (5)	0.607 (17)
H26A	0.582707	0.421581	0.524051	0.210*	0.607 (17)
H26B	0.601579	0.364538	0.559851	0.210*	0.607 (17)
H26C	0.583575	0.311717	0.521119	0.210*	0.607 (17)
C27A	0.5326 (4)	0.3565 (10)	0.5509 (7)	0.116 (5)	0.607 (17)
H27A	0.531256	0.410814	0.530512	0.174*	0.607 (17)
H27B	0.531837	0.300797	0.531433	0.174*	0.607 (17)
H27C	0.517404	0.356308	0.573494	0.174*	0.607 (17)
C28A	0.5613 (5)	0.4454 (14)	0.6142 (8)	0.127 (5)	0.607 (17)
H28A	0.561038	0.502867	0.596054	0.191*	0.607 (17)
H28B	0.545374	0.443543	0.635328	0.191*	0.607 (17)
H28C	0.578106	0.442111	0.632928	0.191*	0.607 (17)
C26B	0.5450 (8)	0.442 (2)	0.6081 (13)	0.124 (6)	0.393 (17)
H26D	0.544557	0.498242	0.588786	0.186*	0.393 (17)
H26E	0.526200	0.423893	0.615929	0.186*	0.393 (17)
H26F	0.555180	0.454079	0.637304	0.186*	0.393 (17)
C27B	0.5906 (6)	0.383 (2)	0.5733 (13)	0.136 (6)	0.393 (17)
H27D	0.592626	0.438095	0.553948	0.203*	0.393 (17)
H27E	0.598362	0.393596	0.604791	0.203*	0.393 (17)
H27F	0.600182	0.331331	0.558197	0.203*	0.393 (17)
C28B	0.5493 (7)	0.3567 (18)	0.5249 (11)	0.124 (5)	0.393 (17)
H28D	0.550629	0.418469	0.511138	0.187*	0.393 (17)
H28E	0.560514	0.313960	0.506354	0.187*	0.393 (17)
H28F	0.530179	0.336420	0.524368	0.187*	0.393 (17)
C29	0.54706 (18)	−0.2407 (5)	0.8176 (3)	0.093 (2)	
H29A	0.566269	−0.221921	0.823193	0.111*	
H29B	0.545963	−0.268918	0.785706	0.111*	
C30	0.5383 (2)	−0.3112 (6)	0.8551 (3)	0.104 (2)	
C31	0.5083 (2)	−0.3401 (6)	0.8486 (4)	0.144 (4)	
H31A	0.503887	−0.389788	0.870762	0.173*	
H31B	0.505428	−0.361475	0.816096	0.173*	
H31C	0.496310	−0.287286	0.854845	0.173*	
C32	0.5418 (2)	−0.2685 (7)	0.9049 (3)	0.138 (3)	
H32A	0.535916	−0.312969	0.928934	0.165*	
H32B	0.530507	−0.212879	0.907327	0.165*	
H32C	0.561026	−0.252564	0.910032	0.165*	
C33	0.5569 (3)	−0.3968 (7)	0.8477 (5)	0.176 (5)	
H33A	0.559224	−0.408183	0.813792	0.211*	
H33B	0.548410	−0.450551	0.862607	0.211*	
H33C	0.574852	−0.385573	0.862193	0.211*	
C34	0.58197 (18)	0.2373 (5)	0.7962 (3)	0.089 (2)	
H34A	0.597884	0.205942	0.810762	0.106*	
H34B	0.567889	0.245701	0.821059	0.106*	
C35	0.59094 (15)	0.3319 (5)	0.7775 (3)	0.080 (2)	
C36	0.6136 (2)	0.3184 (7)	0.7402 (3)	0.132 (3)	
H36A	0.619180	0.378394	0.727680	0.159*	
H36B	0.606604	0.280159	0.714296	0.159*	
H36C	0.629378	0.288067	0.754809	0.159*	
C37	0.56685 (19)	0.3821 (6)	0.7551 (4)	0.128 (4)	
H37A	0.559080	0.344121	0.729744	0.153*	
H37B	0.573139	0.440705	0.741842	0.153*	
H37C	0.552828	0.393729	0.779132	0.153*	
C38	0.60272 (19)	0.3866 (6)	0.8202 (3)	0.117 (3)	
H38A	0.617281	0.350683	0.835249	0.140*	
H38B	0.588102	0.398326	0.843147	0.140*	
H38C	0.610191	0.445232	0.808985	0.140*	
C39	0.52736 (16)	−0.2326 (5)	0.4937 (3)	0.090 (2)	
H39A	0.514780	−0.219931	0.467058	0.109*	
H39B	0.516871	−0.265245	0.518591	0.109*	
C40	0.55100 (17)	−0.2952 (5)	0.4763 (3)	0.089 (2)	
C41	0.57107 (18)	−0.3144 (5)	0.5154 (3)	0.109 (3)	
H41A	0.561516	−0.343427	0.542011	0.130*	
H41B	0.579369	−0.256589	0.525946	0.130*	
H41C	0.585404	−0.355806	0.503775	0.130*	
C42	0.5665 (3)	−0.2467 (8)	0.4363 (4)	0.165 (4)	
H42A	0.581635	−0.285762	0.425598	0.198*	
H42B	0.573753	−0.188060	0.447819	0.198*	
H42C	0.553978	−0.235384	0.409791	0.198*	
C43	0.5379 (2)	−0.3833 (6)	0.4582 (5)	0.170 (5)	
H43A	0.526347	−0.409933	0.483061	0.204*	
H43B	0.552213	−0.427341	0.449472	0.204*	
H43C	0.526616	−0.369519	0.430384	0.204*	
C44	0.4760 (3)	0.3023 (12)	0.6497 (5)	0.172 (4)	
H44	0.467544	0.355100	0.636364	0.207*	
C45	0.4908 (3)	0.3035 (12)	0.6910 (5)	0.171 (4)	
H45	0.492711	0.360552	0.706956	0.205*	
C46	0.5034 (3)	0.2262 (13)	0.7111 (4)	0.160 (4)	
H46	0.514181	0.232330	0.738975	0.191*	
C47	0.5004 (3)	0.1472 (11)	0.6917 (5)	0.176 (4)	
H47	0.508328	0.095312	0.706772	0.211*	
C48	0.4860 (3)	0.1330 (11)	0.6498 (6)	0.183 (4)	
H48	0.484166	0.073479	0.636375	0.220*	

### 1,4-Bis(neopentyloxy)pillar[5]arene Atomic displacement parameters (Å^2^)

**Table:**

	*U*^11^	*U*^22^	*U*^33^	*U*^12^	*U*^13^	*U*^23^
O1	0.134 (4)	0.066 (3)	0.067 (3)	0.022 (3)	−0.031 (3)	−0.005 (2)
N1	0.146 (7)	0.257 (11)	0.138 (7)	−0.022 (8)	−0.024 (6)	0.030 (7)
O2	0.126 (4)	0.061 (3)	0.067 (3)	0.009 (3)	−0.007 (3)	0.008 (2)
O3	0.080 (3)	0.057 (3)	0.092 (3)	0.005 (2)	0.010 (2)	0.016 (2)
O4	0.093 (3)	0.059 (3)	0.062 (3)	−0.011 (2)	0.001 (2)	−0.002 (2)
O5	0.065 (3)	0.064 (3)	0.086 (3)	0.013 (2)	−0.006 (2)	−0.022 (2)
C1	0.051 (4)	0.060 (4)	0.057 (4)	0.002 (3)	0.010 (3)	0.005 (3)
C2	0.070 (4)	0.058 (4)	0.059 (4)	0.008 (3)	−0.001 (3)	0.000 (3)
C3	0.077 (5)	0.059 (5)	0.062 (4)	0.002 (3)	−0.008 (3)	0.013 (4)
C4	0.058 (4)	0.057 (4)	0.050 (4)	0.002 (3)	0.005 (3)	0.004 (3)
C5	0.076 (5)	0.056 (4)	0.057 (4)	0.007 (3)	0.009 (3)	0.001 (3)
C6	0.060 (4)	0.060 (4)	0.060 (4)	0.006 (3)	0.009 (3)	0.016 (3)
C7	0.065 (5)	0.073 (5)	0.060 (4)	−0.003 (3)	−0.009 (3)	0.001 (3)
C8	0.061 (4)	0.063 (4)	0.049 (3)	0.004 (3)	−0.004 (3)	0.000 (3)
C9	0.069 (4)	0.063 (4)	0.055 (4)	0.004 (3)	−0.003 (3)	0.001 (3)
C10	0.069 (4)	0.061 (5)	0.054 (4)	0.001 (3)	−0.005 (3)	0.008 (3)
C11	0.062 (4)	0.064 (4)	0.048 (3)	0.001 (3)	−0.006 (3)	0.000 (3)
C12	0.078 (5)	0.061 (4)	0.057 (4)	0.003 (4)	0.000 (4)	−0.002 (3)
C13	0.077 (4)	0.067 (4)	0.052 (4)	−0.012 (4)	−0.006 (3)	0.005 (3)
C14	0.070 (7)	0.085 (8)	0.059 (6)	−0.001 (6)	0.000	0.000
C15	0.075 (5)	0.060 (4)	0.060 (4)	−0.006 (4)	0.000 (3)	−0.010 (3)
C16	0.063 (5)	0.065 (5)	0.052 (4)	0.011 (3)	0.004 (3)	−0.006 (3)
C17	0.061 (4)	0.061 (4)	0.042 (3)	0.011 (3)	0.002 (3)	0.005 (3)
C18	0.070 (5)	0.065 (4)	0.051 (4)	0.003 (3)	0.003 (3)	0.009 (3)
C19	0.103 (6)	0.061 (5)	0.090 (5)	0.002 (4)	−0.022 (4)	0.002 (4)
C20	0.104 (6)	0.048 (4)	0.118 (7)	0.002 (4)	−0.045 (5)	0.004 (4)
C21	0.179 (12)	0.138 (9)	0.180 (11)	0.024 (8)	−0.001 (9)	0.087 (8)
C22	0.109 (8)	0.116 (7)	0.185 (10)	0.000 (6)	−0.050 (7)	−0.018 (7)
C23	0.180 (10)	0.078 (6)	0.191 (10)	0.028 (6)	−0.069 (8)	−0.030 (7)
C24	0.145 (5)	0.073 (4)	0.095 (5)	0.000 (4)	0.001 (4)	0.012 (4)
C25	0.132 (5)	0.075 (4)	0.104 (5)	−0.008 (4)	−0.007 (4)	0.023 (4)
C26A	0.155 (9)	0.128 (9)	0.138 (10)	−0.014 (8)	0.011 (8)	0.046 (9)
C27A	0.141 (10)	0.076 (7)	0.132 (10)	0.006 (8)	−0.022 (8)	0.038 (7)
C28A	0.156 (13)	0.077 (7)	0.149 (9)	−0.011 (10)	−0.020 (10)	0.022 (7)
C26B	0.154 (13)	0.077 (9)	0.141 (11)	−0.006 (12)	−0.013 (12)	0.021 (9)
C27B	0.145 (10)	0.122 (11)	0.139 (13)	−0.027 (10)	−0.007 (11)	0.021 (11)
C28B	0.155 (11)	0.097 (9)	0.121 (9)	−0.007 (10)	−0.017 (9)	0.034 (9)
C29	0.108 (5)	0.074 (5)	0.097 (5)	0.005 (4)	0.014 (4)	0.014 (4)
C30	0.116 (6)	0.080 (5)	0.115 (5)	0.012 (4)	0.019 (5)	0.034 (4)
C31	0.148 (8)	0.109 (7)	0.176 (9)	−0.031 (6)	0.020 (7)	0.029 (6)
C32	0.153 (8)	0.157 (8)	0.103 (6)	0.002 (7)	0.008 (6)	0.041 (5)
C33	0.191 (10)	0.102 (7)	0.234 (12)	0.054 (7)	0.056 (9)	0.054 (7)
C34	0.107 (6)	0.075 (5)	0.084 (5)	−0.005 (4)	−0.001 (4)	−0.006 (4)
C35	0.078 (5)	0.068 (5)	0.094 (5)	−0.014 (4)	−0.011 (4)	0.001 (4)
C36	0.117 (8)	0.144 (8)	0.136 (8)	−0.043 (6)	0.017 (7)	0.001 (7)
C37	0.113 (7)	0.084 (6)	0.185 (10)	0.002 (5)	−0.044 (7)	0.014 (6)
C38	0.123 (7)	0.095 (6)	0.133 (7)	−0.023 (5)	−0.020 (6)	−0.025 (5)
C39	0.091 (5)	0.084 (5)	0.096 (5)	0.018 (4)	−0.010 (4)	−0.039 (4)
C40	0.095 (5)	0.086 (5)	0.086 (5)	0.032 (4)	−0.015 (4)	−0.033 (4)
C41	0.127 (6)	0.099 (6)	0.100 (6)	0.048 (5)	−0.020 (5)	−0.023 (5)
C42	0.201 (10)	0.179 (9)	0.116 (7)	0.081 (7)	0.047 (7)	0.019 (7)
C43	0.142 (9)	0.121 (7)	0.247 (12)	0.047 (6)	−0.046 (8)	−0.110 (7)
C44	0.135 (8)	0.235 (10)	0.147 (8)	0.009 (8)	0.005 (6)	0.044 (8)
C45	0.149 (9)	0.237 (10)	0.128 (8)	−0.010 (8)	0.015 (6)	−0.002 (7)
C46	0.144 (8)	0.240 (12)	0.095 (7)	−0.031 (8)	−0.021 (6)	0.006 (7)
C47	0.159 (8)	0.236 (10)	0.131 (8)	−0.037 (8)	−0.012 (6)	0.024 (8)
C48	0.165 (9)	0.238 (10)	0.147 (8)	−0.029 (8)	−0.022 (7)	0.021 (8)

### 1,4-Bis(neopentyloxy)pillar[5]arene Geometric parameters (Å, º)

**Table:**

O1—C3	1.374 (7)	C27A—H27A	0.9600
O1—C19	1.409 (7)	C27A—H27B	0.9600
N1—C48	1.357 (16)	C27A—H27C	0.9600
N1—C44	1.445 (17)	C28A—H28A	0.9600
O2—C6	1.382 (7)	C28A—H28B	0.9600
O2—C24	1.405 (9)	C28A—H28C	0.9600
O3—C10	1.388 (7)	C26B—H26D	0.9600
O3—C29	1.412 (8)	C26B—H26E	0.9600
O4—C13	1.393 (7)	C26B—H26F	0.9600
O4—C34	1.410 (8)	C27B—H27D	0.9600
O5—C16	1.369 (7)	C27B—H27E	0.9600
O5—C39	1.412 (7)	C27B—H27F	0.9600
C1—C2	1.369 (8)	C28B—H28D	0.9600
C1—C6	1.386 (8)	C28B—H28E	0.9600
C1—C18	1.524 (8)	C28B—H28F	0.9600
C2—C3	1.392 (8)	C29—C30	1.504 (10)
C2—H2	0.9300	C29—H29A	0.9700
C3—C4	1.376 (8)	C29—H29B	0.9700
C4—C5	1.392 (8)	C30—C31	1.506 (13)
C4—C7	1.527 (8)	C30—C32	1.516 (13)
C5—C6	1.388 (8)	C30—C33	1.524 (12)
C5—H5	0.9300	C31—H31A	0.9600
C7—C8	1.516 (8)	C31—H31B	0.9600
C7—H7A	0.9700	C31—H31C	0.9600
C7—H7B	0.9700	C32—H32A	0.9600
C8—C13	1.375 (8)	C32—H32B	0.9600
C8—C9	1.384 (8)	C32—H32C	0.9600
C9—C10	1.376 (8)	C33—H33A	0.9600
C9—H9	0.9300	C33—H33B	0.9600
C10—C11	1.393 (8)	C33—H33C	0.9600
C11—C12	1.387 (8)	C34—C35	1.507 (9)
C11—C14	1.512 (8)	C34—H34A	0.9700
C12—C13	1.385 (8)	C34—H34B	0.9700
C12—H12	0.9300	C35—C37	1.492 (10)
C14—H144	0.89 (7)	C35—C36	1.510 (11)
C14—H144^i^	0.89 (7)	C35—C38	1.522 (10)
C15—C17^i^	1.380 (8)	C36—H36A	0.9600
C15—C16	1.384 (9)	C36—H36B	0.9600
C15—H15	0.9300	C36—H36C	0.9600
C16—C17	1.391 (8)	C37—H37A	0.9600
C17—C18	1.508 (9)	C37—H37B	0.9600
C18—H18A	0.9700	C37—H37C	0.9600
C18—H18B	0.9700	C38—H38A	0.9600
C19—C20	1.504 (9)	C38—H38B	0.9600
C19—H19A	0.9700	C38—H38C	0.9600
C19—H19B	0.9700	C39—C40	1.519 (10)
C20—C22	1.501 (11)	C39—H39A	0.9700
C20—C23	1.502 (11)	C39—H39B	0.9700
C20—C21	1.518 (13)	C40—C41	1.473 (10)
C21—H21A	0.9600	C40—C43	1.491 (11)
C21—H21B	0.9600	C40—C42	1.501 (12)
C21—H21C	0.9600	C41—H41A	0.9600
C22—H22A	0.9600	C41—H41B	0.9600
C22—H22B	0.9600	C41—H41C	0.9600
C22—H22C	0.9600	C42—H42A	0.9600
C23—H23A	0.9600	C42—H42B	0.9600
C23—H23B	0.9600	C42—H42C	0.9600
C23—H23C	0.9600	C43—H43A	0.9600
C24—C25	1.478 (10)	C43—H43B	0.9600
C24—H24A	0.9700	C43—H43C	0.9600
C24—H24B	0.9700	C44—C45	1.346 (18)
C25—C27B	1.50 (3)	C44—H44	0.9300
C25—C27A	1.513 (17)	C45—C46	1.374 (17)
C25—C26A	1.52 (2)	C45—H45	0.9300
C25—C28B	1.56 (3)	C46—C47	1.257 (16)
C25—C28A	1.59 (2)	C46—H46	0.9300
C25—C26B	1.62 (3)	C47—C48	1.363 (18)
C26A—H26A	0.9600	C47—H47	0.9300
C26A—H26B	0.9600	C48—H48	0.9300
C26A—H26C	0.9600		
			
C3—O1—C19	118.7 (5)	C25—C28A—H28A	109.5
C48—N1—C44	122.6 (13)	C25—C28A—H28B	109.5
C6—O2—C24	119.3 (5)	H28A—C28A—H28B	109.5
C10—O3—C29	117.9 (5)	C25—C28A—H28C	109.5
C13—O4—C34	115.3 (5)	H28A—C28A—H28C	109.5
C16—O5—C39	116.0 (5)	H28B—C28A—H28C	109.5
C2—C1—C6	117.3 (6)	C25—C26B—H26D	109.5
C2—C1—C18	120.9 (6)	C25—C26B—H26E	109.5
C6—C1—C18	121.8 (5)	H26D—C26B—H26E	109.5
C1—C2—C3	122.9 (6)	C25—C26B—H26F	109.5
C1—C2—H2	118.6	H26D—C26B—H26F	109.5
C3—C2—H2	118.6	H26E—C26B—H26F	109.5
O1—C3—C4	117.1 (5)	C25—C27B—H27D	109.5
O1—C3—C2	123.6 (6)	C25—C27B—H27E	109.5
C4—C3—C2	119.3 (6)	H27D—C27B—H27E	109.5
C3—C4—C5	118.9 (6)	C25—C27B—H27F	109.5
C3—C4—C7	122.5 (5)	H27D—C27B—H27F	109.5
C5—C4—C7	118.6 (6)	H27E—C27B—H27F	109.5
C6—C5—C4	120.5 (6)	C25—C28B—H28D	109.5
C6—C5—H5	119.8	C25—C28B—H28E	109.5
C4—C5—H5	119.8	H28D—C28B—H28E	109.5
O2—C6—C1	116.0 (5)	C25—C28B—H28F	109.5
O2—C6—C5	122.9 (6)	H28D—C28B—H28F	109.5
C1—C6—C5	121.1 (6)	H28E—C28B—H28F	109.5
C8—C7—C4	113.0 (5)	O3—C29—C30	109.9 (6)
C8—C7—H7A	109.0	O3—C29—H29A	109.7
C4—C7—H7A	109.0	C30—C29—H29A	109.7
C8—C7—H7B	109.0	O3—C29—H29B	109.7
C4—C7—H7B	109.0	C30—C29—H29B	109.7
H7A—C7—H7B	107.8	H29A—C29—H29B	108.2
C13—C8—C9	117.9 (6)	C29—C30—C31	111.4 (8)
C13—C8—C7	121.2 (6)	C29—C30—C32	109.3 (7)
C9—C8—C7	120.9 (6)	C31—C30—C32	108.8 (8)
C10—C9—C8	121.2 (6)	C29—C30—C33	106.2 (8)
C10—C9—H9	119.4	C31—C30—C33	108.9 (9)
C8—C9—H9	119.4	C32—C30—C33	112.3 (9)
C9—C10—O3	122.4 (6)	C30—C31—H31A	109.5
C9—C10—C11	121.4 (6)	C30—C31—H31B	109.5
O3—C10—C11	116.1 (6)	H31A—C31—H31B	109.5
C12—C11—C10	116.9 (6)	C30—C31—H31C	109.5
C12—C11—C14	121.3 (5)	H31A—C31—H31C	109.5
C10—C11—C14	121.8 (5)	H31B—C31—H31C	109.5
C13—C12—C11	121.5 (6)	C30—C32—H32A	109.5
C13—C12—H12	119.3	C30—C32—H32B	109.5
C11—C12—H12	119.3	H32A—C32—H32B	109.5
C8—C13—C12	121.0 (6)	C30—C32—H32C	109.5
C8—C13—O4	119.8 (6)	H32A—C32—H32C	109.5
C12—C13—O4	119.1 (6)	H32B—C32—H32C	109.5
C11—C14—C11^i^	113.2 (8)	C30—C33—H33A	109.5
C11—C14—H144	109 (5)	C30—C33—H33B	109.5
C11^i^—C14—H144	106 (5)	H33A—C33—H33B	109.5
C11—C14—H144^i^	106 (5)	C30—C33—H33C	109.5
C11^i^—C14—H144^i^	109 (5)	H33A—C33—H33C	109.5
H144—C14—H144^i^	114 (10)	H33B—C33—H33C	109.5
C17^i^—C15—C16	123.0 (6)	O4—C34—C35	111.7 (6)
C17^i^—C15—H15	118.5	O4—C34—H34A	109.3
C16—C15—H15	118.5	C35—C34—H34A	109.3
O5—C16—C15	124.1 (6)	O4—C34—H34B	109.3
O5—C16—C17	116.5 (6)	C35—C34—H34B	109.3
C15—C16—C17	119.3 (5)	H34A—C34—H34B	108.0
C15^i^—C17—C16	117.5 (6)	C37—C35—C34	110.6 (7)
C15^i^—C17—C18	121.0 (6)	C37—C35—C36	109.3 (7)
C16—C17—C18	121.5 (5)	C34—C35—C36	109.0 (7)
C17—C18—C1	112.7 (5)	C37—C35—C38	111.3 (7)
C17—C18—H18A	109.1	C34—C35—C38	107.3 (6)
C1—C18—H18A	109.1	C36—C35—C38	109.3 (7)
C17—C18—H18B	109.1	C35—C36—H36A	109.5
C1—C18—H18B	109.1	C35—C36—H36B	109.5
H18A—C18—H18B	107.8	H36A—C36—H36B	109.5
O1—C19—C20	111.0 (6)	C35—C36—H36C	109.5
O1—C19—H19A	109.4	H36A—C36—H36C	109.5
C20—C19—H19A	109.4	H36B—C36—H36C	109.5
O1—C19—H19B	109.4	C35—C37—H37A	109.5
C20—C19—H19B	109.4	C35—C37—H37B	109.5
H19A—C19—H19B	108.0	H37A—C37—H37B	109.5
C22—C20—C23	107.6 (8)	C35—C37—H37C	109.5
C22—C20—C19	109.9 (7)	H37A—C37—H37C	109.5
C23—C20—C19	108.0 (7)	H37B—C37—H37C	109.5
C22—C20—C21	111.3 (8)	C35—C38—H38A	109.5
C23—C20—C21	109.2 (8)	C35—C38—H38B	109.5
C19—C20—C21	110.7 (7)	H38A—C38—H38B	109.5
C20—C21—H21A	109.5	C35—C38—H38C	109.5
C20—C21—H21B	109.5	H38A—C38—H38C	109.5
H21A—C21—H21B	109.5	H38B—C38—H38C	109.5
C20—C21—H21C	109.5	O5—C39—C40	111.8 (6)
H21A—C21—H21C	109.5	O5—C39—H39A	109.3
H21B—C21—H21C	109.5	C40—C39—H39A	109.3
C20—C22—H22A	109.5	O5—C39—H39B	109.3
C20—C22—H22B	109.5	C40—C39—H39B	109.3
H22A—C22—H22B	109.5	H39A—C39—H39B	107.9
C20—C22—H22C	109.5	C41—C40—C43	111.4 (7)
H22A—C22—H22C	109.5	C41—C40—C42	107.8 (8)
H22B—C22—H22C	109.5	C43—C40—C42	110.3 (8)
C20—C23—H23A	109.5	C41—C40—C39	111.2 (6)
C20—C23—H23B	109.5	C43—C40—C39	106.7 (7)
H23A—C23—H23B	109.5	C42—C40—C39	109.4 (7)
C20—C23—H23C	109.5	C40—C41—H41A	109.5
H23A—C23—H23C	109.5	C40—C41—H41B	109.5
H23B—C23—H23C	109.5	H41A—C41—H41B	109.5
O2—C24—C25	112.8 (7)	C40—C41—H41C	109.5
O2—C24—H24A	109.0	H41A—C41—H41C	109.5
C25—C24—H24A	109.0	H41B—C41—H41C	109.5
O2—C24—H24B	109.0	C40—C42—H42A	109.5
C25—C24—H24B	109.0	C40—C42—H42B	109.5
H24A—C24—H24B	107.8	H42A—C42—H42B	109.5
C24—C25—C27B	102.8 (14)	C40—C42—H42C	109.5
C24—C25—C27A	106.0 (9)	H42A—C42—H42C	109.5
C24—C25—C26A	113.7 (10)	H42B—C42—H42C	109.5
C27A—C25—C26A	109.7 (12)	C40—C43—H43A	109.5
C24—C25—C28B	121.5 (12)	C40—C43—H43B	109.5
C27B—C25—C28B	103.9 (17)	H43A—C43—H43B	109.5
C24—C25—C28A	106.4 (10)	C40—C43—H43C	109.5
C27A—C25—C28A	111.3 (12)	H43A—C43—H43C	109.5
C26A—C25—C28A	109.8 (13)	H43B—C43—H43C	109.5
C24—C25—C26B	108.6 (14)	C45—C44—N1	112.4 (15)
C27B—C25—C26B	108.3 (18)	C45—C44—H44	123.8
C28B—C25—C26B	110.6 (17)	N1—C44—H44	123.8
C25—C26A—H26A	109.5	C44—C45—C46	124.5 (16)
C25—C26A—H26B	109.5	C44—C45—H45	117.7
H26A—C26A—H26B	109.5	C46—C45—H45	117.7
C25—C26A—H26C	109.5	C47—C46—C45	119.5 (14)
H26A—C26A—H26C	109.5	C47—C46—H46	120.3
H26B—C26A—H26C	109.5	C45—C46—H46	120.3
C25—C27A—H27A	109.5	C46—C47—C48	123.8 (17)
C25—C27A—H27B	109.5	C46—C47—H47	118.1
H27A—C27A—H27B	109.5	C48—C47—H47	118.1
C25—C27A—H27C	109.5	N1—C48—C47	117.0 (16)
H27A—C27A—H27C	109.5	N1—C48—H48	121.5
H27B—C27A—H27C	109.5	C47—C48—H48	121.5
			
C6—C1—C2—C3	1.5 (9)	C12—C11—C14—C11^i^	89.1 (5)
C18—C1—C2—C3	−177.4 (6)	C10—C11—C14—C11^i^	−87.5 (5)
C19—O1—C3—C4	−177.8 (6)	C39—O5—C16—C15	22.9 (8)
C19—O1—C3—C2	1.3 (10)	C39—O5—C16—C17	−158.2 (6)
C1—C2—C3—O1	−179.0 (6)	C17^i^—C15—C16—O5	−175.5 (5)
C1—C2—C3—C4	0.2 (9)	C17^i^—C15—C16—C17	5.7 (8)
O1—C3—C4—C5	178.0 (5)	O5—C16—C17—C15^i^	176.4 (5)
C2—C3—C4—C5	−1.2 (9)	C15—C16—C17—C15^i^	−4.7 (7)
O1—C3—C4—C7	−2.4 (9)	O5—C16—C17—C18	−5.6 (8)
C2—C3—C4—C7	178.4 (6)	C15—C16—C17—C18	173.3 (5)
C3—C4—C5—C6	0.5 (9)	C15^i^—C17—C18—C1	92.8 (6)
C7—C4—C5—C6	−179.1 (5)	C16—C17—C18—C1	−85.1 (7)
C24—O2—C6—C1	−177.3 (6)	C2—C1—C18—C17	93.7 (7)
C24—O2—C6—C5	0.5 (9)	C6—C1—C18—C17	−85.2 (7)
C2—C1—C6—O2	175.6 (5)	C3—O1—C19—C20	176.0 (6)
C18—C1—C6—O2	−5.5 (8)	O1—C19—C20—C22	−65.9 (9)
C2—C1—C6—C5	−2.2 (8)	O1—C19—C20—C23	177.1 (7)
C18—C1—C6—C5	176.7 (6)	O1—C19—C20—C21	57.5 (10)
C4—C5—C6—O2	−176.4 (5)	C6—O2—C24—C25	159.9 (7)
C4—C5—C6—C1	1.2 (9)	O2—C24—C25—C27B	−92.6 (17)
C3—C4—C7—C8	−103.6 (7)	O2—C24—C25—C27A	64.5 (13)
C5—C4—C7—C8	76.0 (7)	O2—C24—C25—C26A	−56.1 (15)
C4—C7—C8—C13	−89.9 (7)	O2—C24—C25—C28B	23 (2)
C4—C7—C8—C9	90.4 (7)	O2—C24—C25—C28A	−177.0 (11)
C13—C8—C9—C10	1.5 (8)	O2—C24—C25—C26B	152.8 (15)
C7—C8—C9—C10	−178.8 (5)	C10—O3—C29—C30	156.6 (6)
C8—C9—C10—O3	−176.0 (5)	O3—C29—C30—C31	57.5 (10)
C8—C9—C10—C11	1.5 (9)	O3—C29—C30—C32	−62.7 (9)
C29—O3—C10—C9	17.8 (8)	O3—C29—C30—C33	175.9 (8)
C29—O3—C10—C11	−159.8 (6)	C13—O4—C34—C35	−177.1 (6)
C9—C10—C11—C12	−2.1 (8)	O4—C34—C35—C37	59.2 (9)
O3—C10—C11—C12	175.6 (5)	O4—C34—C35—C36	−61.0 (8)
C9—C10—C11—C14	174.7 (6)	O4—C34—C35—C38	−179.2 (6)
O3—C10—C11—C14	−7.6 (8)	C16—O5—C39—C40	161.0 (6)
C10—C11—C12—C13	−0.4 (8)	O5—C39—C40—C41	57.5 (9)
C14—C11—C12—C13	−177.2 (6)	O5—C39—C40—C43	179.2 (8)
C9—C8—C13—C12	−3.9 (8)	O5—C39—C40—C42	−61.5 (9)
C7—C8—C13—C12	176.3 (5)	C48—N1—C44—C45	2.9 (19)
C9—C8—C13—O4	−179.1 (5)	N1—C44—C45—C46	0.1 (19)
C7—C8—C13—O4	1.2 (8)	C44—C45—C46—C47	−3 (2)
C11—C12—C13—C8	3.4 (9)	C45—C46—C47—C48	3 (2)
C11—C12—C13—O4	178.7 (5)	C44—N1—C48—C47	−3 (2)
C34—O4—C13—C8	−117.8 (6)	C46—C47—C48—N1	0 (2)
C34—O4—C13—C12	66.9 (7)		

Symmetry code: (i) −*x*+1, −*y*, *z*.

### 1,4-Bis(neopentyloxy)pillar[5]arene Hydrogen-bond geometry (Å, º)

π denotes the centroid of the pyridine ring.

**Table:**

*D*—H···*A*	*D*—H	H···*A*	*D*···*A*	*D*—H···*A*
C46—H46···O4	0.93	2.88	3.56 (1)	131
C24—H24*B*···π	0.97	2.99	3.921 (11)	161
C19—H19*B*···N1^i^	0.97	2.97	3.81 (1)	145
C39—H39*B*···N1^i^	0.97	3.17	3.74 (1)	120

Symmetry code: (i) −*x*+1, −*y*, *z*.
